# A Study on the Sense of Parental Competence, Health Locus of Control and Levels of Anxiety, Depression and Somatization in Parents of Children with Type 1 Diabetes: Evidence on a Possible Relationship

**DOI:** 10.3390/jcm13082259

**Published:** 2024-04-13

**Authors:** Ivonne Carosi Arcangeli, Giovanna Celia, Laura Girelli, Chiara Fioretti, Mauro Cozzolino

**Affiliations:** 1 Department of Humanities, Philosophy and Education, University of Salerno, 84084 Fisciano, Italy; lgirelli@unisa.it (L.G.); cfioretti@unisa.it (C.F.); mcozzolino@unisa.it (M.C.); 2 Scupsis—Scuola di Psicoterapia Strategica Integrata Seraphicum, 00142 Rome, Italy; direzione@scupsis.org

**Keywords:** type 1 diabetes, sense of competence, self-efficacy, parents, health locus of control, anxiety, depression, somatization, chronic illness

## Abstract

**Background**: Parents of children with chronic conditions face challenges that go beyond basic care and parenting responsibilities. Parents’ experiences can be influenced by perceived stress, emotional experiences, feelings of helplessness, low sense of self-efficacy, anxiety and depression, reducing their quality of life. It is therefore not surprising that parents of children with chronic illnesses are more likely to experience stress, anxiety and depression than parents of healthy children. A prevalent chronic condition is type 1 diabetes. **Methods**: Parents (31 with children with type 1 diabetes diagnosis and 71 with children without chronic illness) were recruited to complete the measures of the Brief Symptom Inventory-18 (BSI-18), the Parent Health Locus of Control (PHLOC) and Parenting Sense of Competence (PSOC). **Results**: Significant differences in depression and internal locus of control were found; there was a positive correlation between internal LOC and efficacy in both samples; furthermore, there was a negative correlation between somatization and satisfaction in the experimental group. **Conclusions**: The ongoing experiences and challenges faced daily make parents perceive themselves as capable. Active involvement in supporting and managing the needs of child with type 1 diabetes could be a source of empowerment for the parent, contributing to the maintenance of their sense of competence. It is important, therefore, to consider the well-being and perception of the parent at a personal level, regardless of the child’s situation.

## 1. Introduction

According to the definition provided by Stein et al. [1], a chronic pediatric disease can be considered as such if it has an expected or actual duration of 12 months, entails functional limitations and dependence on compensatory mechanisms, and necessitates hospitalizations and/or home care beyond routine treatments. A prevalent chronic condition is type 1 diabetes. Type 1 diabetes (T1DM) stands as one of the most prevalent endocrine diseases among children worldwide [2], with a continually increasing incidence on a global scale. According to a Global Burden of Disease (GBD) study, the number of children affected by diabetes has risen by 64,287 cases from 1990 to 2019 [3]. This form of diabetes can manifest at any age, but it is particularly aggressive in young individuals [4]. This study aims to investigate two main questions: “What is the relationship between levels of depression, anxiety, and somatization, the sense of parenting competence, and the health locus of control in parents of children with type 1 diabetes? What are the differences in these constructs between parents of children with type 1 diabetes and parents of children without chronic illness?” Parents of children with chronic conditions face challenges that go beyond basic care and parental responsibilities. Parents’ experiences can be influenced by perceived stress, emotional experiences, feeling of impotence, low sense of self-efficacy, anxiety and depression, reducing their quality of life. It is therefore not surprising that parents of children with chronic illness are more likely to feel stress, anxiety and depression than parents of healthy children. A thorough analysis of the psychosocial profile, well-being and mental functioning of parents proves extremely advantageous, as a chronic pathological condition always entails parental involvement and adaptation to the emerging situation [5]. For parents, the diagnosis of diabetes represents a particularly stressful experience that changes the overall structure of the family system, impacting the parent’s psychological functioning and overall well-being. Specific challenges for parents in managing T1D in younger children have been elucidated [6,7]. At the time of diagnosis, parents of preschool children must shoulder complete responsibility for all daily management activities, encompassing the monitoring of blood glucose levels, regulation of nutrition and physical activity, and insulin administration [6,7]. The process of managing a child with Type 1 diabetes is described by parents as a profoundly transformative journey, necessitating a complete reorientation of one’s life and daily activities. This new reality, marked by the constant attention required to ensure the child’s well-being, inevitably triggers a profound shift in the family’s dynamics and routines. The magnitude of these changes is significant, impacting not only the parents but also the child and the broader family unit in various ways. This impact manifests as increased stress, anxiety and depression [8,9,10,11]. Furthermore, it is important to consider various additional factors when discussing the experiences of parents of children with chronic illnesses. These factors encompass the sense of efficacy, satisfaction and health locus of control. Coined by Bandura, self-efficacy is an individual’s confidence in their ability to perform a specific behavior [12], and it has been highlighted that an increase in patients’ self-efficacy is associated with various health improvements, including adherence to medical prescriptions, acquisition of health knowledge, reduction in disease activity, and adoption of positive health behaviors in various populations and diseases [13,14]. In the case of parents who have children with Type 1 diabetes, it has been observed that symptoms of stress, anxiety and depression are often linked to a reduced sense of self-efficacy [10,11]. Specifically, the majority of research in this area has centered on the perceived self-efficacy of parents in managing their child’s diabetes, rather than examining the broader concept of parental self-efficacy in a more comprehensive manner [15,16]. Despite this, the precise role of parental self-efficacy in relation to health outcomes associated with diabetes in young children is not yet fully understood. However, the existing literature suggests that evaluating the level of parental self-efficacy can be valuable for clinical purposes, as it can help to inform the development of effective interventions and ensure that appropriate support is provided to parents in managing their child’s condition. In particular, assessing parental self-efficacy can provide insights into the specific challenges and barriers that parents may face in caring for a child with diabetes, as well as the strategies that they may use to overcome these challenges. By taking a more holistic view of the caregiving experience, it can better understand the complex interplay of factors that can impact the health and well-being of both parents and children [17]. Another factor that is worth taking into account is that of satisfaction, which refers to the pleasure and fulfillment that parents derive from their role as parents [18]. A growing body of research has highlighted that lower levels of parental satisfaction are often linked to dysfunctional parenting practices and behavioral problems in children [19,20]. Another relevant factor is the health locus of control, which refers to an individual’s beliefs about their personal influence on their health. This concept includes both the internal locus of control, which reflects the belief that one’s actions and thoughts can impact health outcomes, and the external locus of control, which reflects the perception that health outcomes are determined by external entities such as healthcare professionals, God, or chance [21,22]. Given the crucial role that parents play in promoting and managing their children’s health, it is essential to assess their health locus of control, particularly in cases where children have chronic illnesses. By improving parents’ health locus of control [23], we can enhance the adherence to interventions and ultimately improve health outcomes for both parents and children. In particular, assessing parents’ health locus of control can provide insights into the specific beliefs and attitudes that may impact their ability to manage their child’s health, as well as the strategies that they may use to promote their own health and that of their child [24]. To conduct this research, parents of children with type 1 diabetes were recruited through the “AGD Umbria” Association, which focuses on supporting families affected by this condition, and parents of children without any chronic or psychiatric conditions in the general population. To optimize data collection, measurements were digitized using an online questionnaire. This innovative approach enables greater participation and ease of completion for participants, ensuring efficient and accurate data collection. An academic platform was used, ensuring data security through the adoption of appropriate protective measures to prevent unauthorized access or loss of information. Specific measures were adopted to assess the aforementioned constructs: the Parent Health Locus of Control, previously used in various studies examining the locus of control of parents of children with chronic illnesses, to evaluate parents’ beliefs about their child’s health; the Parenting Sense of Competence, used to assess parental satisfaction and perceived competence; and the Brief Symptom Inventory-18 used to evaluate psychological distress. 

## 2. Materials and Methods

### 2.1. Sample

The sample consisted of two groups of parents of children. The experimental group comprised *n* = 31 parents of children with type 1 diabetes, while the control group comprised *n* = 71 parents of children without chronic and/or psychiatric conditions. Parents of children with type 1 diabetes were recruited in collaboration with an association called “Associazione Giovani Diabetici Umbria (AGD Umbria)”. After presenting the research work, contacts were established through the association to engage the parents by explaining the objectives of the study and requesting participation through informed consent. The control group was recruited from the general population, similarly explaining the research objectives and requesting participation through informed consent. The exclusion criterion for control group was the presence of chronic and/or psychiatric conditions in the children. The online questionnaire included a specific mandatory question: “Does your child have chronic and/or psychiatric conditions?” In case of an affirmative answer, the online questionnaire closed and did not allow further completion.

The mean age of experimental group participants was 46.33 (S.D. = 6.22); *n* = 22 participants were women and *n* = 9 participants were men. The mean age of control group participants was 41.10 (S.D. = 6.76); *n* = 64 participants were women and *n* = 7 participants were men (Table 1). Participants were asked to complete an online questionnaire; the online questionnaire collected information on sociodemographic data and psychological variables. For the group of parents of children with type 1 diabetes, there were questions relating to the onset of the disease, any technological devices used and the possible presence of t1 diabetes in the family.

All subjects gave their informed consent for inclusion before participating in the study. The questionnaires were anonymous to ensure confidentiality and reliability of the data.

All procedures in this study were performed in accordance with the ethical standards of the research committee of the Italian Association of Psychology (AIP) and with the 1964 Helsinki Declaration and its subsequent amendments. No further approval was required.

### 2.2. Measures

All measures were digitized and made accessible to participants from online forms via a link shared across academic platforms.

Before further data analysis, the normality of the data distribution in each scale was checked. The internal consistency of each scale was examined using Cronbach’s alpha [25].

The Brief Symptom Inventory-18 (BSI-18) [26]. The BSI-18 is composed of 18 items answered on a five-point Likert scale (0 = not at all, 1 = a little, 2 = moderately, 3 = somewhat, 4 = extremely) to evaluate psychological distress. Participants are asked to report how much they have suffered from that problem in the past seven days. The BSI-18 is a condensed version of the Symptoms Checklist-90-Revised (SCL-90-R) [27] and the Brief Symptom Inventory (BSI) [28]. The BSI-18 captures three components of psychological distress: somatization, depression and anxiety. Finally, an overall global psychological distress score is calculated. Already employed in an Italian sample by Tremolada et al. [29]. The Cronbach’s α of the depression scale for this study was 0.67. The Cronbach’s α of the anxiety scale for this study was 0.85. The Cronbach’s α of the somatization scale for this study was 0.70.

The Parent Health Locus of Control [22] is a questionnaire composed of 30 items answered on a 6-point Likert scale (1 = strongly disagree and 6 = strongly agree agreement) to assess a parent’s beliefs about a child’s health. The questionnaire evaluates the child’s beliefs (6 items), i.e., the extent to which parents think that their child directly controls his or her health, the divine (4 items), i.e., the parents’ beliefs about the importance of God in influencing health of the child, of destiny (5 elements), i.e., an index of the extent to which parents believe that the state of health of their child is mainly a question of luck, of the media (4 elements), i.e., a measure of how much parents believe that all the media, such as TV, magazines and books, can directly influence the well-being of their children, of parents (6 items), i.e., an evaluation of how much parents feel the main responsibility for the health of their children, and professional influences (5 items), i.e., an estimation of the extent to which parents think health care providers monitor their child’s health. Participants are asked to express their degree of agreement or disagreement with each statement reported in the questionnaire. For each dimension, an average score is calculated by summing all the values of each statement/item belonging to the subscales and therefore varies from 1 to 6 for each subscale. The categories ‘parent’ and ‘child’ refer to an internal HLOC, whereas health professionals, the media, God and fate identify an external HLOC. In the Italian version, 2 of the 30 items were eliminated as they were not appropriate for mothers of children under 3 years of age (my child can decide to live a healthy and safe life, and my child’s reading influences his well-being) [24]. The Cronbach’s α of the child scale for this study was 0.79. The Cronbach’s α of the parent scale for this study was 0.84. The Cronbach’s α of the destiny scale for this study was 0.82. The Cronbach’s α of the divine scale for this study was 0.90. The Cronbach’s α of the professionals scale for this study was 0.71. The Cronbach’s α of the media scale for this study was 0.86.

The Parenting Sense of Competence (PSOC) [30] consists of 17 items on a 6-point Likert scale ranging from “strongly disagree” to “strongly agree” to measure parental satisfaction and perceived competence in the parenting role [31]. It includes two subscales, efficacy and satisfaction. The original version of the PHLOC was forward-translated into Italian by a bilingual researcher and then back-translated by another bilingual researcher. The two versions were compared according to back-translation techniques [32]. The Cronbach’s α of the efficacy scale for this study was 0.75. The Cronbach’s α of the satisfaction scale for this study was 0.74. 

## 3. Results

### 3.1. Psychological Distress

The one-way analysis of variance of three subscales of the Brief Symptom Inventory-18 (BSI-18) between the two groups showed a significant differences in depression subscales (Table 2). The depression levels were high in the group of parents with children diagnosed with type 1 diabetes [*F*(1100) = 5.81; *p* = 0.018].

### 3.2. Parental Health Locus of Control

The one-way analysis of variance of six subscales of the Parent Health Locus of Control between the two groups showed a significant differences in three subscales (Table 3). These subscales were child, media and professionals. The scores on the child subscale [*F*(1100) = 28.82; *p* = 0.000] and the professionals subscale [*F*(1100) = 19.65; *p* = 0.000] were higher in the group of parents with children diagnosed with type 1 diabetes. However, the scores on the media subscale were higher in parents of healthy children [*F*(1100) = 4.61; *p* = 0.034]. 

The one-way analysis of variance of the internal subscale and external subscale of the Parent Health Locus of Control between the two groups showed a significant differences in the internal subscale (Table 4). The scores for the internal subscale were higher in the group of parents with children diagnosed with type 1 diabetes [*F*(1100) = 6.42; *p* = 0.013].

### 3.3. Parenting Sense of Competence

No significant difference were found between the two groups for the Parenting Sense of Competence scale (PSOC).

During the analysis of correlations, the following correlations were obtained:

- Experimental group.

### 3.4. Association between BSI-18 and PSOC (Experimental Group)

The result of a correlation analysis between the BSI-18 subscale and PSOC subscale showed a negative correlation between the somatization subscale of BSI-18 and satisfaction subscale of PSOC *r*(31) = −0.464, *p* < 0.01. 

### 3.5. Association between PHLOC and PSOC (Experimental Group)

The results of a correlation analysis between the PHLOC subscale and PSOC subscale showed a positive correlation between the child subscale and efficacy subscale *r*(31) = 0.37, *p* < 0.05, and between the parent subscale and efficacy subscale *r*(31) = 0.517, *p* < 0.01, and a negative correlation between the destiny subscale and satisfaction subscale *r*(31) = −0.442, *p* < 0.05. Furthermore, there was a positive correlation between the internal LOC and efficacy subscale *r*(31) = 0.546, *p* < 0.01. 

### 3.6. Association between BSI-18 and PHLOC (Experimental Group)

The results of a correlation analysis between the BSI-18 subscale and PHLOC subscale did not show a significant correlation.

- Control group.

### 3.7. Association between BSI-18 and PSOC (Control Group)

The results of a correlation analysis between the BSI-18 subscale and PSOC subscale showed a negative correlation between the somatization subscale and satisfaction subscale *r*(71) = −0.255, *p* < 0.05 and a negative correlation between the depression subscale and satisfaction subscale *r*(71) = −0.349, *p* < 0.01.

### 3.8. Association between PHLOC and PSOC (Control Group)

The results of a correlation analysis between the PHLOC subscale and PSOC subscale showed a positive correlation between the child subscale and efficacy subscale *r*(71) = 0.429, *p* < 0.01 and a negative correlation between the destiny subscale and satisfaction subscale *r*(71) = −0.261, *p* < 0.05. Furthermore, there was a positive correlation between the internal LOC and efficacy subscale *r*(71) = 0.425, *p* < 0.01.

### 3.9. Association between BSI-18 and PHLOC (Control Group)

The results of a correlation analysis between the BSI-18 subscale and PHLOC subscale showed a positive correlation between the anxiety subscale and destiny subscale *r*(71) = 0.237, *p* < 0.05 and a negative correlation between the anxiety subscale and professionals subscale *r*(71) = −0.253, *p* < 0.05.

## 4. Discussion

The purpose of this study was to investigate a potential relationship between levels of depression, anxiety and somatization, as well as the sense of efficacy, satisfaction and locus of control in parents of children with type 1 diabetes. Additionally, we aimed to examine the differences in these constructs in parents of children with type 1 diabetes compared to those of parents with children without a chronic pathology. This result may have been influenced by an uneven distribution between mothers and fathers. Comparisons between the samples revealed that parents of children with type 1 diabetes exhibited higher levels of depression than parents of children without chronic diseases. Consistent with scientific literature, the prevalence of depression is higher among parents of children with type 1 diabetes compared to the general population, suggesting the need for screening and interventions for depression in parents of children with type 1 diabetes [33]. Other significant differences between the two samples emerged regarding the health locus of control. This study highlighted that parents of children with type 1 diabetes exhibited a predominantly internal locus of control compared to parents of healthy children. This result may have been influenced by active involvement in the daily management of their children’s condition. In the context of pediatric diabetes studies, parental locus of control has been studied in relation to the glycemic control of the child, demonstrating that children with optimal control had parents with a predominant internal locus of control compared to those with suboptimal glycemic control [34]. Regarding the sense of parental competence, no significant differences were found between the two groups. The results of this study suggest that the sense of parental competence is not compromised by the presence of the child’s pathology, as might be expected. Hence, it is conceivable to hypothesize that the ongoing experiences and daily challenges encountered lead parents to perceive themselves as competent. It can be hypothesized that given the crucial role parents play in managing both the illness and the health of their child, engagement in the child’s health management may contribute to enhancing parental efficacy. Active involvement in supporting and addressing the needs of the sick child could serve as a source of empowerment for the parent, thereby sustaining their sense of competence. Even the correlations among the constructs examined showed interesting results in the group of parents of children with type 1 diabetes. The results demonstrated that the internal locus of control positively correlates with the sense of parental efficacy. This result might suggest that parents of children with type 1 diabetes who perceive a greater internal control over their children’s health also tend to feel more effective in their parenting role, and vice versa. This positive correlation could imply that strengthening parents’ sense of internal control might be an important area to consider in support programs for parents of children with type 1 diabetes, as it could positively influence their perception of parenting efficacy and, consequently, their overall well-being. Furthermore, a negative correlation emerged between levels of somatization and levels of parental satisfaction. This result might suggest that in parents of children with type 1 diabetes, the experience of somatic symptoms could be associated with lower parental satisfaction, and vice versa. This could indicate that perceived physical discomfort might negatively influence the overall well-being of parents and their perception of the quality of their parenting role. Therefore, it could be beneficial to adopt a mind–body integration approach both in analyzing the needs of these parents and in developing intervention strategies to improve their overall well-being and parental satisfaction. Therefore, it is acknowledged that the emotional and psychological well-being of the parent constitutes a pivotal element in the equation of managing the child’s illness. This study suggest that is also important to consider the well-being and perception of the parent on a personal level, independently of the child’s situation, focusing on analyzing how the parent perceives themselves in relation to these aspects. This study presents several limitations. Firstly, the participant pool is characterized by a low number, and there is an uneven distribution between mothers and fathers. The overrepresentation of mothers may imply that the findings are more reflective of the maternal experience than the paternal experience. Therefore, it would be valuable to explore potential differences based on parental roles. Another limitation pertains to the utilization of the Parenting Sense of Competence instrument. During the experimental phase, a new version of the test was validated and published, addressing certain critical issues in the original version [35]. Consequently, future perspectives could aim to increase the sample size to ensure a balanced representation of parental roles and further delve into the considered variables. Additionally, it might be appropriate to incorporate the updated version of the questionnaire to obtain more precise results. This could prompt further studies to enable a better understanding of the relationship between parental sense of competence, the health locus of control, and psychological distress. The need for these additional studies also arises from the potential future use of such evidence in the development of intervention programs.

## 5. Conclusions

The experiences and challenges faced daily may lead parents to perceive themselves as competent. Therefore, this study suggests that the sense of parental competence may not be compromised by the presence of the child’s condition, as one might expect. Active involvement in supporting and managing the needs of a child with type 1 diabetes could be a source of empowerment for the parent, contributing to the maintenance of their sense of competence. The importance of considering the health locus of control and psychological distress is also emphasized. Further research could allow for a deeper exploration of these constructs, enabling an analysis in relation to the parental role played.

## Figures and Tables

**Table 1:** Description of participants.

Variable	Experimental Group (*n* = 31)	Control Group (*n* = 71)
Female	22	64
Male Age, y, mean (SD)	9 46.33 (6.22)	7 41.18 (6.76)

**Table 2:** Analysis of variance for Brief Symptom Inventory-18 (BSI-18).

	Experimental Group		Control Group			
	Mean	SD	Mean	SD	*F*-Value	*p*-Value
BSI_Depression	5.09	4.30	3.42	2.63	5.81	0.018

**Table 3:** Analysis of variance for Parent Health Locus of Control (PHLOC).

	Experimental Group		Control Group			
	Mean	SD	Mean	SD	*F*-Value	*p*-Value
PHLOC_Child	4.38	0.87	3.27	0.99	28.82	0.000
PHLOC_Media	3	1.33	3.61	1.32	4.61	0.034
PHLOC_Professional	4.34	0.78	3.49	0.93	19.65	0.000

**Table 4:** Analysis of variance Internal subscale for Parent Health Locus of Control (PHLOC).

	Experimental Group		Control Group			
	Mean	SD	Mean	SD	*F*-Value	*p*-Value
Internal LOC	4.19	0.64	3.82	0.69	6.42	0.013

## Data Availability

The data presented in this study are available on request from the corresponding author.
